# Editorial: Novel theranostic agents for precision therapeutics

**DOI:** 10.3389/fphar.2024.1407366

**Published:** 2024-04-17

**Authors:** Shahid Karim, Nasir Ali Siddiqui, Mohammad Imran Khan, Muhammad Wahajuddin, Shariq Syed

**Affiliations:** ^1^ Department of Clinical Pharmacology, Faculty of Medicine, King Abdulaziz University, Jeddah, Saudi Arabia; ^2^ Department of Pharmacognosy, College of Pharmacy, King Saud University, Riyadh, Saudi Arabia; ^3^ Research Center, King Faisal Specialist Hospital and Research Center, Jeddah, Saudi Arabia; ^4^ Institute of Cancer Therapeutics, School of Pharmacy and Medical Sciences, Faculty of Life Sciences, University of Bradford, Bradford, United Kingdom; ^5^ School of Pharmacy, Anjuman Islam Kalsekar Technical Campus, Navi Mumbai, India

**Keywords:** diagnostic, delivery platforms, theranostics (combined therapeutic and diagnostic technology), small molecules, nanotechnology

The integration of novel theranostic agents signifies a significant progression in the realm of personalised medicine, particularly in the dynamic domain of precision medicines. The term “theranostics” was introduced by John Funkhouser (Kelkar and Reineke, 2011). It was introduced to describe scientific progress in selectively targeting particular diseases and integrating diagnostic and therapeutic functions into a single substance, resulting in a potential therapeutic framework that encompasses diagnosis, medication administration, and monitoring of treatment efficacy (Pratihar et al., 2023). This editorial explores the increasingly prominent subject of theranostic agents and their capacity to transform the provision of healthcare to patients.

Theranostic agents encompass a diverse range of chemicals that are developed for the dual purpose of disease diagnosis and targeted therapeutic interventions. Clinicians are able to gain a comprehensive understanding of disease processes and achieve precise treatment by employing advanced imaging techniques such as positron emission tomography (PET), single-photon emission computed tomography (SPECT), and magnetic resonance imaging (MRI), in conjunction with therapeutic substances (Shrivastava et al., 2019; Crisan et al., 2022).

The primary emphasis of drug treatment has always been on patient-centered care. The transition from universal medicine, sometimes known as “one medicine fits all,” to personalised medicine has significantly transformed drug therapy by offering tailored drug therapy based on individual drug response (Jeelani et al., 2014; Shrivastava et al., 2019). Theranostics, a remarkable platform that integrates diagnostic and therapeutic approaches, has the potential to offer treatment regimens that are tailored to individual patients, thereby enhancing prognoses and fostering a closer integration between diagnosis and therapy (Crisan et al., 2022; Su et al., 2024). Theranostics in personalised medicine provide patients with the appropriate medication at the optimal dosage, hence enhancing the safety and efficacy of pharmacotherapy.

One notable example of theranostic progress can be observed in the field of oncology, whereby the use of radiolabeled peptides and antibodies enables the detection of tumor-specific biomarkers and the precise delivery of radiation to malignant cells. According to Fendler et al. (2023), the use of 68Ga-labeled prostate-specific membrane antigen (PSMA) ligands has yielded notable advancements in the management of prostate cancer (Fendler et al., 2023).

These advancements include enhanced precision in staging and improved therapeutic decision-making, resulting in heightened efficacy and reduced occurrence of adverse effects. The advent of nanotechnology has facilitated the development of versatile theranostic nanomaterials capable of simultaneously detecting, imaging, and treating diseases at the molecular level (Shrivastava et al., 2019; Ayuso et al., 2022; Burkett et al., 2023). The utilisation of bioactive natural substances is a viable route for theranostic applications due to the potential for focussed therapy. Nevertheless, additional investigation is required to improve the composition of these compounds, better their capacity for absorption inside the human body, and assess their effectiveness and safety in clinical environments. In their study, Rehman et al. conducted the synthesis of zinc chromium vanadate nanoparticles, which were subsequently investigated for their potential antibacterial and anticancer characteristics. The structural and morphological aspects of the nanoparticles were elucidated through a comprehensive characterisation. The potential of synthetic nanoparticles in eliminating waterborne *Entererobacteriaceae* and combating cancer has been demonstrated. Ovarian cancer necessitates novel therapies, and Alharbi et al. are investigating the synergistic effects of mangiferin and curcumin. The synergistic activation of the PI3K/Akt/mTOR pathway by both phytochemicals enhances therapeutic efficacy, hence diminishing the need for excessive dosage, adverse effects, and drug resistance. The utilisation of computational drug design enables the advancement and improvement of theranostic medications by means of generating, refining, and tailoring adaptable molecules and drug delivery systems for therapeutics.

In their study, Alhakamy et al. introduced a new neo-tanshinlactone-chalcone hybrid that demonstrates a high affinity for binding to TNF-α in docking tests. Notably, TNF-α is associated with MCF-7 cancer cell lines and increases the expression of aromatase to promote the growth of breast cancer cells in the outer layer of the breast. Possible lead compounds for the treatment of breast cancer.

Suhail et al.’s computational analysis highlights the ability of flavonoids to inhibit PI3Kγ, which encourages additional research on combining traditional pharmacology with innovative treatment methods such as PROTACs.

Suhail et al.’s study investigated the stereo-selectivity of catechins in inhibiting EGFR kinase in both the wild-type and L858R mutant. The results of computational analysis indicate that all stereoisomers, including the well researched catechin (−)-EGCG, have a binding affinity towards the ATP-binding site, hence potentially inhibiting the activity of EGFR kinase. Gallated catechins exhibited superior inhibition of EGFR compared to non-gallated catechins, demonstrating intriguing binding propensities. The stereoisomers that exhibit the highest dock scores and binding energies with wild-type EGFR are (−)-CG, (−)-GCG, (+)-CG, and (−)-EGCG. In order to assess the dynamic behaviour and stability of the top-ranked catechin (−)-CG and the extensively researched (−)-EGCG with EGFR kinase, molecular dynamics simulations were conducted for a duration of 100 nanoseconds. This study enhances our comprehension of the impact of stereoisomers on inhibitory potential, hence facilitating the selection of stereoisomers for improved drug effectiveness.

The innovative therapeutic agent for acute renal damage was introduced by Gumbar et al. The presence of triterpene saponins known as Gymnemic acid was detected in the leaf of *G. sylvestre*, a plant that has been extensively utilised in Ayurvedic medicine. The aforementioned compound has demonstrated advantageous effects in ameliorating acute renal failure and exhibits the capacity to mitigate the oxidative stress induced by gentamicin.

In the current epoch of precision medicine, it is imperative to acknowledge the challenges and ethical considerations associated with the utilisation of theranostics. Securing authorization from regulatory bodies, guaranteeing cost-efficiency, and assuring equitable availability of cutting-edge technologies are substantial obstacles that necessitate cooperative endeavours from all healthcare participants.

In essence, the integration of diagnostics and medicines is the pinnacle of precision medicine, signifying a substantial shift from broad-based interventions to individualised patient care. The profound impact of innovative theranostic agents on the transformation of healthcare delivery is indisputable, notwithstanding the challenges associated with attaining extensive clinical integration.
